# Case report: Psychosis with catatonia in an adult man: a presentation of neurosarcoidosis

**DOI:** 10.3389/fpsyt.2024.1276744

**Published:** 2024-03-04

**Authors:** Griet Van Hoye, Barbara Willekens, Stephanie Vanden Bossche, Manuel Morrens, Filip Van Den Eede

**Affiliations:** ^1^ Collaborative Antwerp Psychiatric Research Institute (CAPRI), Faculty of Medicine and Health Sciences, University of Antwerp, Antwerp, Belgium; ^2^ Department of Neurology, Antwerp University Hospital, Edegem (Antwerp), Belgium; ^3^ Laboratory of Experimental Hematology, Vaccine & Infectious Disease Institute (VAXINFECTIO), Faculty of Medicine and Health Sciences, University of Antwerp, Antwerp, Belgium; ^4^ Translational Neurosciences Research Group, Faculty of Medicine and Health Sciences, University of Antwerp, Antwerp, Belgium; ^5^ Department of Radiology, Antwerp University Hospital, Edegem (Antwerp), Belgium; ^6^ AZ Sint-Jan Bruges, Department of Radiology, Bruges, Belgium; ^7^ Scientific Initiative of Neuropsychiatric and Psychopharmacological Studies (SINAPS), University Psychiatric Centre Duffel, Duffel, Belgium; ^8^ Department of Psychiatry, Antwerp University Hospital, Edegem (Antwerp), Belgium

**Keywords:** psychosis, catatonia, neurosarcoidosis, case report, diagnosis

## Abstract

**Introduction:**

Sarcoidosis is a multisystem non-caseous granulomatous disease of unknown origin with predominant lung involvement and a variable clinical course. Although rare, neuropsychiatric manifestations such as confusion, problems in orientation, memory dysfunction, delusions, hallucinations and catatonia can be presenting features of sarcoidosis with nervous system involvement, also known as neurosarcoidosis.

**Case description:**

We present a 39-year-old man with acute-onset vertigo, balance problems and confusion quickly developing delusions, hallucinations, catatonic symptoms and suicidal behaviour. Symptoms appeared to be a manifestation of neurosarcoidosis.

**Diagnostic assessment:**

The differential diagnosis of psychosis is broad and should include pertinent auto-immune disorders, paraneoplastic, oncologic, metabolic, and neurodegenerative disorders. Basic systemic screening should include blood and urinary tests, a chest X-ray, brain CT scan and ECG. If neurosarcoidosis is suspected, an MRI of the brain with contrast and lumbar puncture are most appropriate. Multidisciplinary collaboration is essential to arrive at a correct diagnosis and effective management of the patient.

**Discussion:**

Despite the large number of sarcoidosis and psychosis studies, the etiology and pathogenesis of both illnesses remain incompletely understood. A common inflammatory etiopathological pathway has been postulated.

**Conclusions:**

Clinicians should consider organic causes when confronted with a middle-aged patient experiencing a first psychotic episode with an atypical onset, catatonic features, or dysfunction in orientation and/or memory, a complete lack of a positive familial psychiatric history and/or an atypical response to (psycho)pharmacological treatment.

## 1 Introduction

Sarcoidosis or Besnier-Boeck-Schaumann disease is a multisystem disease of unknown origin with predominant lung involvement and a variable clinical course, in which non-caseous granulomatous infiltration of affected organs is the pathological hallmark (1, 2). It is a global disease, with a prevalence of about 4.7 - 64 in 100 000, and an incidence of 1.0 - 35.5 in 100 000 per year (1). Particularly women from African-American and to a lesser degree Northern-European origins are considered to be most susceptible to developing sarcoidosis (1, 3). The incidence peaks between 30 and 60 years of age (2).

Extrapulmonary manifestations of sarcoidosis have been described in 15–25% of patients (2). Central and peripheral nervous systems can be involved, in which case the diagnosis neurosarcoidosis is made (3). Central nervous system (CNS) involvement occurs in 5% to 10% and most frequently concerns cranial neuropathy, but also aseptic meningitis, hydrocephalus, myopathy, peripheral neuropathy, CNS parenchymatous disease, and seizures have been noted (1, 3). The prognosis of neurosarcoidosis is highly variable and ranges from a single episode of illness, through relapsing–remitting courses, to progressive disability (1, 3).

Although rare, neuropsychiatric manifestations such as confusion, dysfunction in orientation and/or memory, auditory and/or hallucinations, delusions and catatonia can be presenting features of neurosarcoidosis (4–10). To emphasize the importance of distinguishing primary from secondary forms of psychosis, a case of a 39-year-old male patient is discussed below.

## 2 Case description

A 39-year-old man of European ancestry first presented at the emergency department of a local hospital in Flanders, Belgium with acute-onset vertigo, balance problems and nightly confusion. The patient had a medical history of pulmonary sarcoidosis seven years prior for which he was prescribed hydroxychloroquine 200 mg per day as maintenance treatment. However, he had stopped taking the tablets a couple of months before presentation, without consulting or informing his treating physician. He had been diagnosed with diabetes mellitus, for which he didn’t adhere to prescribed treatments, resulting in poor glycemic control. His and his family’s psychiatric histories were negative for psychosis. Nonetheless, three years earlier he had been seen at the department of psychiatry for anxiety symptoms. At the time though, personal antecedents of childhood trauma were deemed grounds for a referral to a psychotherapist.

## 3 Diagnostic assessment

The patient was admitted for neurological evaluation. Laboratory assessments, lumbar puncture, CT and MRI of the brain were executed. Neurological examination was normal except for cognitive signs such as fluctuating problems with attention and confabulations. No alcohol or illicit drug use were reported, and the urinary drug screening was negative, as well as serum alcohol level. Standard laboratory tests (including thyroid function tests) were normal, except for a CRP of 35.6 mg/L (increasing to 69.2 mg/L) and raised glucose with a HbA1c of 7,4%. Four days later he was admitted to the psychiatric ward because of paranoid delusions and auditory hallucinations. Lorazepam 2.5mg was administered orally with paradoxical effect, aggravating the delirious symptoms, agitation and delusions, and was therefore discontinued. Instead, olanzapine 10mg, trazodone 100mg and lormetazepam 2mg were administrated once daily at night. Five days after admission in the psychiatric ward, he was urgently transferred to the hospital’s intensive care unit because of acute hypoxemic hypercapnic respiratory failure. Psychopharmacological treatment was paused. Chest X-ray demonstrated bilateral parenchymatous infiltrates and pleural fluid compatible with bilateral pneumonia. Antimicrobial treatment with clarithromycin and amoxicillin was started empirically. In bronchoalveolar lavage (BAL) and endotracheal aspiration (ETA) samples, H. influenza and coronavirus were detected. After intubation and sedation, he was transferred to our university hospital for further diagnostic workup.

During his stay, he presented with a variety of psychotic symptoms, in particular agitation, confusion, auditory and visual hallucinations, and paranoid and bizarre delusions (poisoning, acrophobia while in bed). Also, impulsive self-harm was observed, i.e. the patient cutting his arm with a torn soda can. After evaluation however, no persistent parasuicidal behavior was retained. During psychiatric evaluation with the Bush Francis Catatonia Rating Scale Dutch version (BFCRS-N), 7 items were retained (11, 12). Notable clinical findings included stupor (1), catalepsy (2), psychomotor agitation (4), rigidity (1), gegenhalten (3) and autonomic instability (2) such as tachycardia (110 bpm) and tachypnoea. Olanzapine 5 to 10mg seemed to aggravate these symptoms and was stopped for this reason. Neuroleptic malignant syndrome seemed unlikely given that blood pressure was normal, there was no fever, and serum creatine kinase, leukocytes, electrolytes and kidney function remained within the normal range. To treat catatonic symptoms, he was again started on lorazepam 1mg three times daily as needed, since treatment with benzodiazepines is first choice for catatonia and a new paradoxical reaction on delirious symptoms may not occur at lower dosages (13, 14).

**Figure 1:**
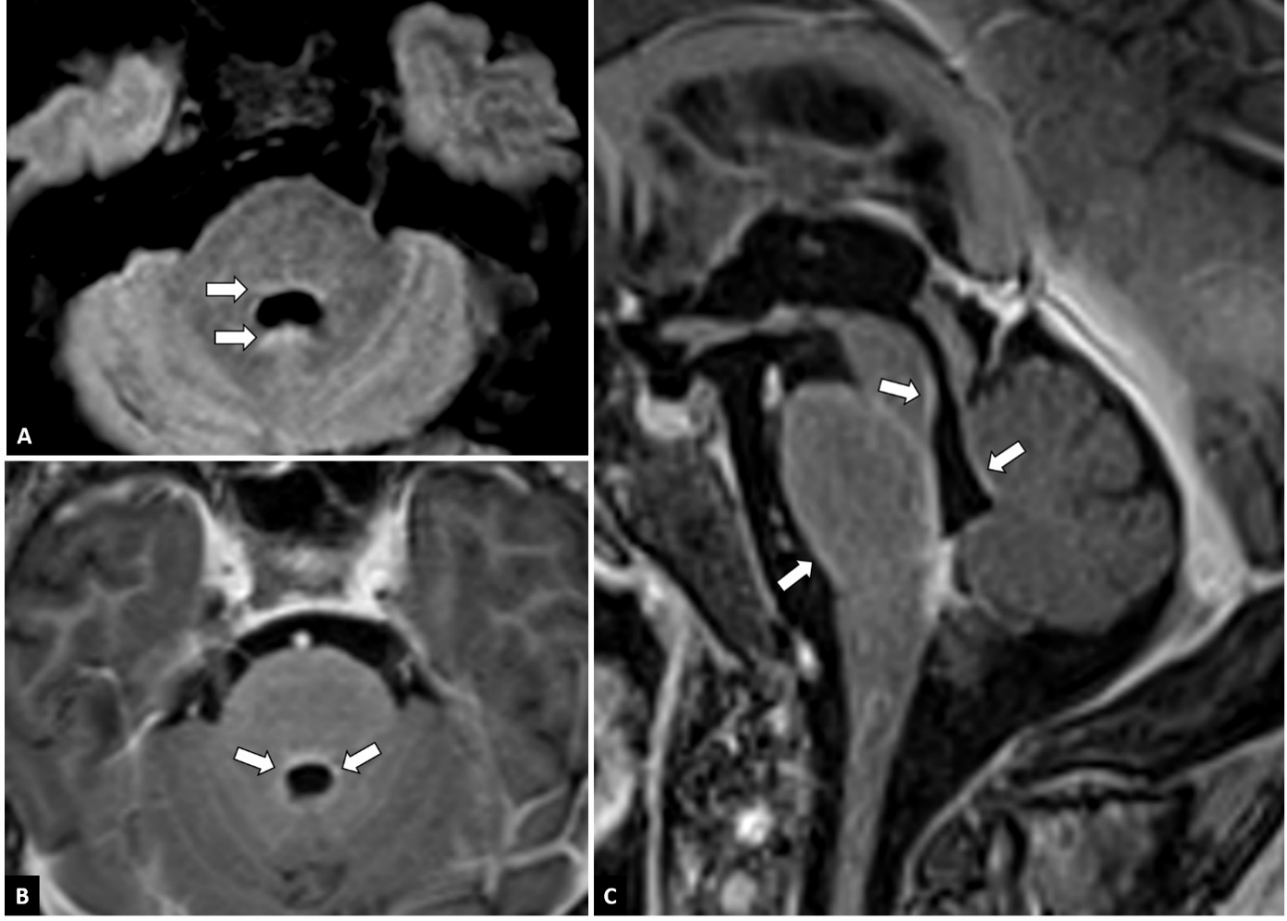
MRI features. Axial FLAIR-weighted images at the level of the fourth ventricle **(A)** show discrete FLAIR-hyperintense signals around the fourth ventricle (arrows). On axial **(B)** and sagittal **(C)** post-contrast T1-weighted images of the fossa posterior, leptomeningeal contrast enhancement can be seen around the brainstem and in the walls of the fourth ventricle (arrows).

On lumbar puncture, there was an elevated opening pressure (37 cmH_2_0 and 45 cmH_2_0 with Valsalva). Cerebrospinal fluid (CSF) analysis demonstrated a raised white blood cell count (5/µL) and elevated protein (100 mg/dL) during treatment with amoxicillin-clavulanate. The latter was administrated because of a urinary infection with culture positive for ciprofloxacine-resistent E. coli. Antibiotic treatment was later on switched to ceftriaxone and co-trimoxazole when the culture was known. CSF gram stain was negative. The CSF cultures for bacteria and mycobacteria did not show any organisms. Polymerase Chain Reaction (PCR) for Herpes Simplex Virus, Epstein-Barr Virus, Cytomegalovirus, Toxoplasma gondii and Hepatitis B and C were negative. CSF cytology and flow cytometry were unremarkable (13). Anatomopathological examination of CSF did not show malignant cells. Panels for paraneoplastic antibodies; antinuclear antibodies (ANA), antiribonucleoprotein autoantibodies (Anti-RNP), anti-small nuclear ribonucleoprotein antibodies (snRNP), antibodies to SSa antigen (Anti-Ro-52 and anti-Ro-60), antibodies to SSb antigen (Anti-La), Anti-Scl-70, Anti-Jo-1, Anti-CENP-B, Anti-DFS70 and antineutrophil cytoplasmic antibodies (ANCA) and autoimmune encephalitis antibodies; anti-NMDA receptor antibodies (anti-NMDAR); anti-smooth muscle antibodies (Anti-SMA), anti-mitochondrial antibodies (Anti-AMA), anti-liver-kidney microsomal antibodies (Anti-LKM) and anti-tissue transglutaminase IgA (Anti-tTg-IgA) remained negative on serum and on CSF. Meanwhile PET-CT showed diffuse lung abnormalities and enlarged mediastinal and hilar lymph nodes consistent with pulmonary sarcoidosis; no tumoral masses were detected. Electroencephalography (EEG) showed no signs of epileptic activity and a normal alpha rhythm although many artefacts were present. Contrast-enhanced magnetic resonance imaging (MRI) demonstrated infratentorial leptomeningeal enhancement, most notably around the brain stem and in the fourth ventricle walls (**Figure 1**). Angiotensin-converting enzyme (ACE) was normal in serum and CSF (23 U/L and 9 U/L).

Taking into account the patient’s history of pulmonary sarcoidosis with pathologic confirmation of systemic granulomatous disease 7 years prior, the clinical presentation and MRI features typical of granulomatous inflammation and the rigorous exclusion of other causes, a diagnosis of probable neurosarcoidosis was made following the 2018 Neurosarcoidosis Consortium Consensus criteria (15). This patient developed catatonia and delirium with persistent psychotic symptoms in the context of a urinary tract infection, bilateral pneumonia due to sarcoidosis and a probable diagnosis of neurosarcoidosis **Figure 1**.

Treatment with intravenous methylprednisolone 1g per day for 3 days was initiated, followed by oral prednisolone 60mg daily for a minimum duration of one month. His symptoms improved significantly over the course of the following few days. The CRP dropped to 7 mg/L. Vertigo, agitation, confusion, delusions and hallucinations disappeared completely. Mild depressive symptoms with a Beck depression inventory-2 score of 17 remitted to a score of 8 in the months after stabilization. Symptoms of anxiety remained, meeting the criteria of generalized anxiety disorder (GAD) and specific phobias (claustrophobia and acrophobia), however these disorders preceded the current condition and disease. Behavioral therapy was recommended and initiated. Prednisolone 60mg was tapered down slowly over the course of one year and azathioprine 150mg daily was started as maintenance treatment. We have successfully monitored the patient’s progress for 5 years since. An MRI with gadolinium contrast, conducted 6 months after the initial glucocorticoid treatment, revealed a significant decrease in contrast capture. The previously noted T2 signal increase in the mesiotemporal region on both sides maintained its distribution pattern but showed a slightly lower signal intensity. No new T2 signal abnormalities were observed, and the limited enhancement at the base of the fourth ventricle remained unchanged, with no new areas of discoloration.

**Figure 2:**
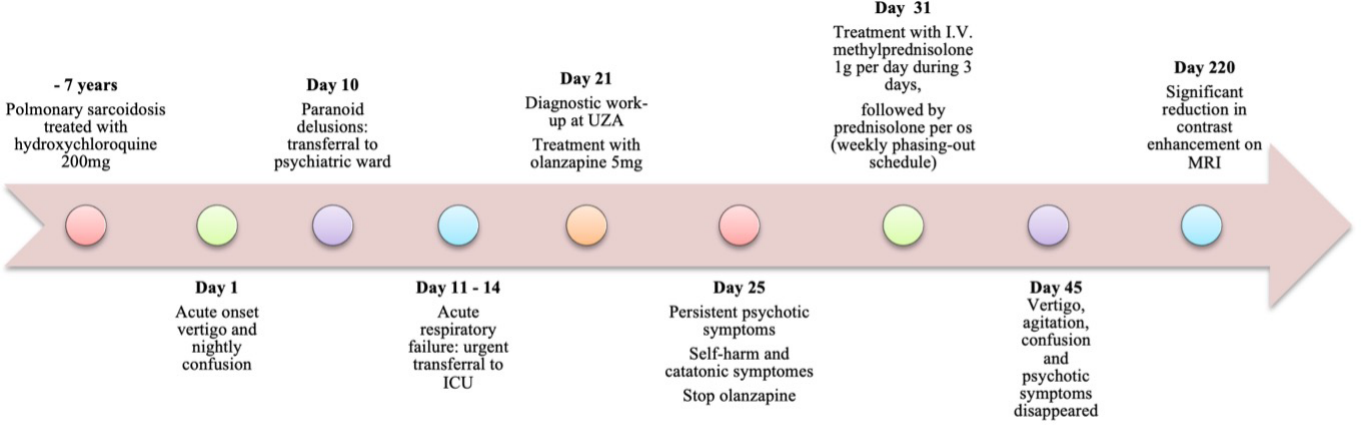
Timeline of key events related to neurosarcoidosis.

A subsequent MRI of the brain, scheduled 3 years later, indicated a new small lacunar infarction in the periventricular left frontal region. There was a reduction in signal abnormalities in the left external capsule. However, there were no further progressive changes, and the limited enhancement at the bottom of the fourth ventricle remained unchanged. The lacunar infarction was asymptomatic and likely had a microvascular origin, considering the patient’s cardiovascular risk profile. Considering the decreased lesions observed on MRI, the stable neurological condition, and recurring infections, the maintenance treatment with azathioprine was reduced to 50 mg once daily. As of today, 5 years after the initial presentation, the patient is in good health, with no reported neurological symptoms **Figure 2**.

## 4 Discussion

When patients present with symptoms of psychosis and/or delirium, an underlying CNS pathology should always be considered in the differential diagnosis (16). An atypical age at onset, any focal or localized psychiatric or neurological symptoms, catatonia, deficits in orientation or memory, a complete lack of a positive familial psychiatric history, an atypical response to (psycho)pharmacological treatment and an atypical clinical course may point towards a non-psychiatric underlying disorder (16).

The differential diagnosis of psychosis is broad and should include pertinent auto-immune disorders, paraneoplastic, oncologic, metabolic and neurodegenerative disorders (4). In such cases, multidisciplinary collaboration is essential to arrive at a correct diagnosis and effective management of the patient (4, 16). Basic systemic screening should include blood and urinary tests, a chest X-ray, brain CT scan, and ECG. If neurosarcoidosis is suspected, an MRI of the brain with contrast and lumbar puncture are most appropriate (16). CSF findings are not specific for neurosarcoidosis, but, if deemed safe, CSF analysis should be considered in cases of suspected neurosarcoidosis to establish the presence of intrathecal inflammation and rule out infectious and neoplastic processes (15). The CSF opening pressure can be elevated (15). Frequent CSF abnormalities include a predominantly mononuclear pleocytosis, elevated protein, low glucose, elevated IgG index, and oligoclonal bands, none of which are specific but provide important evidence for active inflammation (15). Biomarker angiotensin-converting enzyme (ACE) has been used for the diagnosis of sarcoidosis; however, the test is insufficiently specific and sensitive in serum and/or CSF to be of use (15). The proposed consensus diagnostic criteria provide definitions for possible, probable, and definite neurosarcoidosis. The presence of granuloma in central nervous system biopsy increases the level of confidence of the diagnosis of sarcoidosis, but even pathologic identification of granulomas is not 100% definitive (15). In addition, if inflammation is present in a pathologic specimen but granulomas are absent, the patient does not meet the criteria for definite but can be considered to have possible or probable if the clinical circumstances otherwise support these assignments (e.g., presence or absence of granuloma in systemic biopsy) (15).

In sarcoidosis psychiatric comorbidity is common, although psychiatric symptoms are a rare first presentation and psychiatric effects are not well established in the recent neurology literature (7). About 20% to 66% of those with neurosarcoidosis were likely to display psychiatric symptoms, with 25% having a major depressive episode, 6.3% a panic disorder, 6.3% bipolar disorder, 5% generalized anxiety disorder and 1.3% obsessive-compulsive disorder according to the MINI Plus inventory (10, 17, 18).

In the literature, several rare cases have already been published depicting a similar disease course, wherein neurosarcoidosis induces delirium and/or psychotic symptoms in conjunction with catatonia (4–7, 9, 10). Wide-ranging neurological features were recorded including headaches, unsteadiness/incoordination, visual failure, diplopia, fatigue, nausea and vomiting, limb sensory disturbance, seizures, memory impairment, somnolence, walking difficulty, ocular pain, facial weakness, facial sensory loss/pain, hemi-motor and visual-field loss, optic atrophy, papilledema, monoparesis, depression, dysphasia, intention tremor, emotional lability/personality change, deafness, tinnitus and vertigo (7). Although DSM-5 criteria for catatonia secondary to medical conditions exclude comorbid catatonia and delirium, there is a growing literature describing their frequent co-presentation (14, 19). According with these authors and others catatonia scholars, catatonia and catatonia/delirium are underdiagnosed in inpatient wards and should be routinely assessed in patients with an altered mental status (13, 14, 19). For the complete resolution of catatonia, it must be treated simultaneously with its etiology and the possible complications that it may present (13).

The goal of disease-modifying treatment in neurosarcoidosis is to reduce or prevent organ system damage from harmful effects of granulomatous inflammation (3). Treatments focused on symptom management and rehabilitation are also important in the comprehensive care of patients with neurosarcoidosis (3). Neurosarcoidosis is primarily treated with high-dose corticosteroids (Prednisone 0.25–1 mg/kg/d PO and/or Methylprednisolone 1,000 mg/d x 3–5d IV), where careful attention is warranted because of their known dose-related risk of psychiatric symptoms, which can mimic the psychotic symptoms of neurosarcoidosis (1, 3, 4). However, this was not the case in our patient. More evidence is needed regarding the effects of adjunctive symptomatic treatment with antipsychotics in patients with neurosarcoidosis (4). Close clinical and radiologic follow-up is important when tapering glucocorticoids given frequent recurrence of disease activity on steroid withdrawal (3). Methotrexate (10–20 mg once per week orally or intramuscularly) or Azathioprine (50–200 mg per day), in this order, is the preferred second-line therapy for corticosteroid-resistant sarcoidosis or as a corticosteroid-sparing drug (3). Leflunomide (10–20 mg per day), cyclophosphamide (50–150 mg per day), mycophenolate mofetil (500–3000 mg per day) and hydroxychloroquine (200–400 mg/day) are third-line alternatives (1, 3). Several (multi-institutional) studies reported a favorable clinical response in 77% to 96% of patients treated with TNF-alpha antagonist infliximab (3). Infliximab appears capable of inhibiting the formation of granulomas in sarcoidosis and inducing apoptosis via complement-dependent and antibody-dependent cytotoxicity (3). Other TNF antagonists, such as adalimumab, may also be effective in neurosarcoidosis (1, 3).

Despite the large number of sarcoidosis and psychosis studies, the etiology and pathogenesis of both illnesses remain incompletely understood. Inflammatory processes and an altered immune response have been postulated in the pathogenesis in psychosis, sarcoidosis and other CNS inflammatory and auto-immune disorders (20). A common blunted cellular immune response and an over-activated type-2 response is described, especially in the later stages in sarcoidosis and psychosis (4). And while the type-2 response is typically anti-inflammatory, IL-6, a pro-inflammatory cytokine, is a product of activated monocytes and has been referred to as a marker of the type-2 immune response (4). IL-6, IL-8, and TNF-α concentrations have shown to be elevated with the kynurenine pathway in schizophrenia (21). Pro-inflammatory cytokines can stimulate the activity of indoleamine 2,3-dioxygenase and lead to tryptophan deficiency and thus a reduction in the production of 5HT and melatonin, with a shift to the production of kynurenine and other neurotoxic tryptophan-derived metabolites (21). These higher values could then explain the white and grey matter degeneration in schizophrenia and in sarcoidosis patients in granuloma formation, with the possible development of fibrosis in the CNS, assuming an etiological link in pathophysiology between sarcoidosis and psychosis (4).

## 5 Conclusion

Patients with sarcoidosis have a higher risk of psychiatric comorbidities, while those with neurosarcoidosis can present with psychiatric symptoms caused by meningoencephalitis, mimicking primary psychiatric disorders. A common inflammatory etiopathological pathway has been postulated for the two disabling diseases. Clinicians should hence consider organic causes when confronted with a middle-aged patient experiencing a first psychotic episode with an atypical onset, catatonic features or dysfunction in orientation and/or memory, a complete lack of a positive familial psychiatric history and/or an atypical response to (psycho)pharmacological treatment.

## 6 Patient perspective

This case is a clear example of poor treatment adherence, resulting in very serious complications. The patient himself was deeply affected by the entire course, feeling the intensity and severity of it, and experiencing a sense of guilt and worries. His spouse was highly discontented and attributed the blame to the patient, especially considering the numerous psychosocial problems that this course of events caused for the family.

## Data availability statement

The original contributions presented in the study are included in the article/supplementary material. Further inquiries can be directed to the corresponding author.

## Ethics statement

Written informed consent was obtained from the individual(s) for the publication of any potentially identifiable images or data included in this article.

## Author contributions

GV: Investigation, Writing – original draft, Writing – review & editing. BW: Investigation, Writing – review & editing. SV: Investigation. Writing – review & editing. MM: Writing – review & editing. FV: Investigation, Supervision, Writing – review & editing.
